# Study on the influence of periodic roof fracturing on the mechanical disturbance characteristics of coal and gas outburst behavior in the mining face

**DOI:** 10.1371/journal.pone.0337026

**Published:** 2026-02-13

**Authors:** Chunhua Zhang, Ziyue Chen, Xin Wu, Jingyu Ma, Jinquan Chen

**Affiliations:** 1 College of Safety Science and Engineering, Liaoning Technical University, Fuxin, Liaoning, China; 2 Key Laboratory of Mine Thermodynamic Disasters and Control of Ministry of Education(Liaoning Technical University), Huludao, Liaoning, China; China University of Mining and Technology, CHINA

## Abstract

To elucidate the stress evolution characteristics of coal and the gas-dynamic response mechanism during periodic roof fracturing in longwall mining, the 3DEC numerical simulation software was employed to investigate the distribution characteristics of the abutment pressure at the instant of roof fracturing and elastic rebound. Based on the coupling relationship between abutment pressure and gas pressure, the gas occurrence characteristics in the mining face were further analyzed. The results indicate that, with increasing mining advance, the stress distribution after roof fracturing—affected by the roof suspension length and elastic rebound behavior—differs significantly from conventional patterns. As the mining face advances, the length of the overlying hard roof increases, and the elastic rebound behavior at the moment of roof failure reduces the vertical stress on the coal seam, thereby forming a stress-relief zone (Zone II) ahead of the working face. Using the COMSOL Multiphysics software for simulation, it was concluded that the elastic rebound at the moment of roof failure, in conjunction with the cyclic pressurization process, jointly determines the timing characteristics of coal energy release, which has a direct dynamic control effect on the initiation and eruption of gas outbursts. The stress perturbations caused by roof elastic rebound not only alter the mechanical structure and fracture evolution of the coal body, but also influence the occurrence and flow state of gas, thereby controlling the conditions and dynamic intensity of coal and gas outbursts. It provides a new theoretical basis for the prevention and control of gas outbursts and roof-related disasters.

## 1. Introduction

With the continuous extraction of underground coal resources, the overlying strata lose the support of the coal seam and undergo movement, resulting in a redistribution of tangential stress in front of the working face and within the surrounding rock of the roadway. This redistribution can generally be divided into stress-relief zones and stress-concentration zones. For roadways adjacent to goafs in the upper section, the surrounding rock stress is often affected by the superposition of longitudinal and lateral abutment pressures, which makes it more prone to severe strata pressure manifestations such as large-scale roof collapse, coal spalling, and rock bursts. When thick and hard key strata exist in the overburden, the manifestations of strata pressure become even more intense, greatly increasing the likelihood of gas outbursts and significantly aggravating the hazards, thereby posing a serious threat to the safe and efficient operation of coal mines. Therefore, an in-depth investigation into the intrinsic relationships among the spatial structure and movement laws of the overlying strata, the dynamic response of abutment pressure, and the state of gas occurrence is of fundamental importance for the prevention and control of roof collapse and gas-related dynamic disasters. Accurate quantitative analysis of the distribution and evolution characteristics of abutment pressure can not only help reveal the failure mechanisms of surrounding rock under dynamic pressure but also provide theoretical and technical guidance for the rational layout, support design, and safe, efficient mining of roadways.

In the fields of coal mining and geotechnical engineering, extensive research has been conducted on the formation mechanisms, dynamic evolution laws, and monitoring and prediction methods of abutment pressure. These studies have produced substantial achievements and have provided valuable guidance for surrounding rock control and support optimization. Dou et al. [1] proposed the impact pressure intensity-weakening and shock-reduction theory, which effectively mitigates impact ground pressure disasters by reducing the strength and impact tendency of loose coal and rock masses. Fu Y et al. [2] proposed a method for predicting the bearing capacity of key blocks based on regression calculation formulas by analyzing the changes in the force on key blocks, providing an effective theoretical basis for evaluating roof stability. Shi J et al. [3] found that changes in the support resistance conditions adjacent to the tunnel significantly affect the fracture location of the roof, further highlighting the important role of support measures in maintaining roof stability. In addition, with the increasing mining depth, both the mechanical behavior of the roof and the characteristics of gas flow undergo significant changes. This study aims to deeply analyze the evolution characteristics of coal seam stress and the dynamic response mechanism of gas during the periodic roof fracture process using 3DEC numerical simulation software. Kang H et al. [4] systematically analyzed the geomechanical properties of coal seams and their impact on roof control, proposing a ground pressure control technique for deep mining conditions. Zhang J et al. [5], based on elastic thin plate theory, established a mechanical model for roof movement, revealing the impact mechanism of cantilever structures on roof failure and gas release. Most existing research focuses primarily on the impact of single factors on roof stability, lacking a systematic analysis of the roof's elastic rebound behavior and periodic stress disturbances on gas dynamic responses. Dong F et al. [6] and Gao R et al. [7] revealed, through experimental and numerical simulations, the influence of roof stress disturbances on overlying rock fracture evolution, but the coupled effects between stress evolution and gas flow have not been fully explored. Xie H et al. [8]Through experimental testing and mining mechanical simulation, the mechanical response of coal samples from depths greater than 1000 m was studied, analyzing the stress and deformation characteristics of coal and rock bodies during mining, and revealing the response patterns of deep coal and rock bodies under complex mining conditions. Gao M et al. [9], using UDEC discrete element simulations, explored the relationship between the fractal dimension of borehole walls and the distribution of advanced support pressure during face advancement, validating the effectiveness of the fractal dimension in reflecting stress distribution characteristics. Significant progress has also been made in the study of rock damage characteristics and failure mechanisms under dynamic stress. He M et al. [10] conducted dynamic unloading experiments under true triaxial conditions, combining full-wave AE spectral analysis to reveal the damage process of limestone under dynamic stress. Hua A et al. [11] simulated the stress release process through triaxial compression tests, noting that when the initial axial stress exceeds the uniaxial compressive strength of the rock, a decrease in confining pressure tends to cause rock failure, providing new insights into the formation mechanisms of pressure-relief fracturing. Islam MR et al. [12] analyzed the stress redistribution caused by coal mining and its impact on strata and seepage, revealing the changing trends in stress contours during coal seam mining, and proposed that this trend, when further intensified in multi-seam mining, could lead to roof collapse incidents. Breakthroughs have also been made in the research on support and energy absorption. Fu Q et al. [13] proposed a novel pressure-relief and energy absorption system composed of a combined blasting and constant-resistance energy absorption support, based on theoretical analysis and numerical simulation. This method effectively reduces the surrounding rock stress in the tunnel, shortens the length of the suspended roof in mined-out areas, and enhances roof stability. Zhuang J et al. [14] simulated microseismic events to study the impact of mining speed on the roof stress transfer process, revealing the coupling relationship between coal seam blasting risk, advancing speed, and stress redistribution. Wang Q et al. [15], in response to the severe impacts of roof collapse during fully mechanized top-coal caving in thick coal seams, proposed the PREA reinforcement mechanism, proving that it significantly reduces surrounding rock stress levels and ensures tunnel safety and stability. In recent years, significant achievements have also been made in the study of coal and rock body mechanical effects and stress environments ahead of the mining face. Cheng G et al. [16] studied the spatiotemporal evolution characteristics of overlying rock deformation during mining, finding that overlying rock deformation undergoes a dynamic evolution process accompanied by the generation, extension, and closure of fractures. Gao Y et al. [17] proposed the directional roof split blasting (DRSB) technique, which effectively weakens stress concentration in adjacent areas while enhancing roof fracturing effects, providing new ideas for stress control in deep mining conditions. Wang C et al. [18], starting from the constitutive model of rock failure, discussed the characteristics of stress release and energy dissipation in rock during failure, providing important theoretical insights for the study of rock fracture mechanisms. Khan M et al. [19] used Long Short-Term Memory (LSTM) models to predict pressure peaks, revealing the pattern of pressure increase in coal and rock bodies, providing new technological means for mine disaster prediction and early warning. Additionally, Cheng X et al. [20], through theoretical analysis and numerical simulations, explored the impact of lithology on the stress environment ahead of the mining face and studied the mechanical effects of coal and rock bodies under different protective layer mining modes. The results indicated that, under the same mining conditions, a reduction in lithology strength leads to a decrease in the pressure peak at the front, providing important supplementation to the theory of pressure-relief mining in protective layers. In deep coal seam mining, the variation characteristics of electrical parameters (EP) have also become a focal point of research. Niu Y et al. [21] conducted field tests to study the relationship between EP response and outburst risk, finding that the yellow zone of EP response can identify most outburst-prone areas, while the red zone corresponds to regions with higher risk levels. This research provides new methods for the precise identification and prevention of dynamic disasters in deep coal mine strata. Furthermore, Kong X et al. [22] revealed, through dynamic experiments, the damage process of gas-bearing coal bodies in complex mining environments. The study showed that, with increasing impact load, the stress waveform of the rock body exhibits a sinusoidal characteristic, revealing the dynamic disaster mechanism triggered by impact loads. In terms of coal and gas outburst early warning, Li B et al. [23] proposed a novel early warning model, providing an effective means to improve disaster early warning efficiency and safety during coal mining operations. In roof mechanical state analysis, Peng X et al. [24] studied the deflection characteristics of the roof in a highly inclined protective layer in a mine in western Henan, based on elastic thin plate theory. They found that the displacement and fracture development of rock layers could effectively alleviate protective layer pressure and provide channels for gas migration and discharge. Zhang J et al. [25] proposed an analysis method for evaluating the mechanical state of roof strata in underground longwall mining, with calculation results verified by ten independent tests, demonstrating high accuracy and feasibility, providing new ideas for roof management in coal mines. Boothukuri V et al. [26] successfully solved the overburden pressure problem through a combination of geological research, support design, and construction practices, and discussed the influence of mining face width, burial depth, and main roof thickness on roof control behavior. This research provides important engineering references for roof management. Ji S et al. [27] simplified the rock layer to a beam model on an elastic foundation and discussed the impact of support schemes and coal layer deformation on rock behavior, proposing that enhancing support capacity could effectively improve roof stability.

However, as coal mining depth continues to increase, the applicability of existing research findings has become increasingly limited under varying geological and mining conditions, especially in terms of monitoring and predicting abutment pressure distribution. This limitation hinders effective engineering responses, causing difficulties in coal pillar design, support parameter determination, and face layout, and has become a major constraint on safe and efficient mining operations. In coal seams prone to outbursts, previous research on roof fracturing and stress disturbance during mining has primarily focused on strata pressure behavior and control, such as pressure prediction, roof support, and rockburst analysis. Due to the extensive disturbance range of the working face, gas-bearing coal ahead of the face experiences repeated stress perturbations from alternating roof movement and fracturing, leading to continuous evolution of its mechanical and energy states. In localized zones, these dynamic effects can further intensify under the influence of roof fracturing. When the accumulated energy exceeds the resistance of the fragmented coal near the face, outbursts are likely to be triggered. Therefore, this study reveals a new abutment pressure distribution pattern, emphasizing the formation mechanism of a secondary stress-relief zone driven by roof elastic rebound. Through discrete element numerical simulation, the spatiotemporal influence of periodic roof fracture and elastic rebound on stress evolution ahead of the working face is quantitatively characterized. By establishing a mechanistic linkage among stress redistribution, fracture evolution, and gas response, the dynamic triggering mechanism of coal and gas outbursts induced by roof fracture is elucidated, providing theoretical guidance for the prevention and control of roof-related gas dynamic disasters.

## 2. Mechanical model and governing equations of roof fracture

### 2.1. Mechanical model of the mining working face

**Fig 1:**
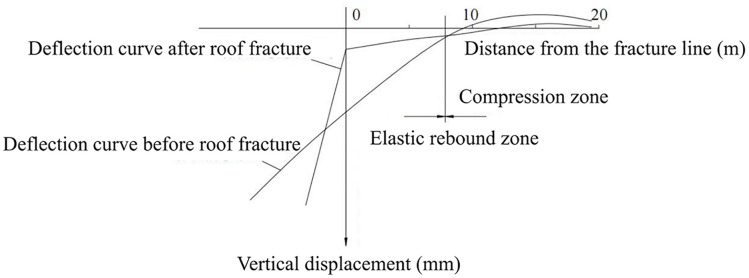
Dynamic disturbance mechanical model of periodic roof fracture.

The study of the disturbance characteristics (rebound compression) induced by the periodic fracture of the main roof essentially investigates the variation pattern of roof deflection throughout the entire mining area before and after each periodic break. As the longwall working face continuously advances, the range of the overlying strata affected by mining disturbance progressively expands. To analyze the evolution of roof deflection under different fracture lengths, degrees, and stages of development, a mechanical model of the periodic fracture of the main roof structure is established, as illustrated in Fig 1.

### 2.2. Structural equation of the roof

The deflection function of the main roof in the mining area, denoted as *ω₀(x, y)*, satisfies the following partial differential equation [28]:


$$\frac{\partial^{\text{4}}\omega_{\text{0}}(x,y)}{\partial x^{\text{4}}}+\text{2}\frac{\partial^{\text{4}}\omega_{\text{0}}(x,y)}{\partial x^{\text{2}}\partial y^{\text{2}}}+\frac{\partial^{\text{4}}\omega_{\text{0}}(x,y)}{\partial y^{\text{4}}}=\frac{\text{1}}{D}q$$ (1)

where *D* denotes the flexural rigidity of the main roof.

Outside the mining area (excluding the fracture lines), the deflection function of the main roof, *ω(x, y)*, satisfies the following partial differential equation:


$$\frac{\partial^{\text{4}}\omega (x,y)}{\partial x^{\text{4}}}+\text{2}\frac{\partial^{\text{4}}\omega (x,y)}{\partial x^{\text{2}}\partial y^{\text{2}}}+\frac{\partial^{\text{4}}\omega (x,y)}{\partial y^{\text{4}}}=\frac{-k\omega (x,y)}{D}$$ (2)

where:


$$D=\frac{E{\text{h}}^{\text{3}}}{\text{12}(\text{1}-\mu^{\text{2}})}$$ (3)

Where *E* is the elastic modulus of the main roof (GPa), *h* is the thickness of the main roof (m), and *μ* is Poisson’s ratio.

### 1.3. Gas distribution in the working face

Under the original geological conditions, the roof remains in a state of energy equilibrium, while the weak structural bodies formed by primary fractures and tectonic activities exist in a sub-equilibrium state. When subjected to mining-induced disturbances, deformation and fracture propagation occur within the excavation space, generating additional dynamic energy. The sub-equilibrium state is first disrupted, leading to an instantaneous release of accumulated energy that can trigger roof-related dynamic disasters. Based on the analysis of stress and deformation characteristics of thick and hard roof strata, an energy accumulation equation for the surrounding rock is established as follows:


$$W_{\text{s}}=\frac{\text{1}}{\text{2}E}\left[\sigma_{\text{1}}^{\text{2}}+\sigma_{\text{2}}^{\text{2}}+\sigma_{\text{3}}^{\text{2}}-\text{2}\nu (\sigma_{\text{1}}\sigma_{\text{2}}+\sigma_{\text{2}}\sigma_{\text{3}}+\sigma_{\text{3}}\sigma_{\text{1}})\right]$$ (4)

Where *E* is the elastic modulus of the rock layer (GPa), *σ₁* is the maximum principal stress, *σ₂* is the intermediate principal stress, and *σ₃* is the minimum principal stress (MPa).

## 2. Numerical simulation

### 2.1. Model construction and parameter settings

**Fig 2:**
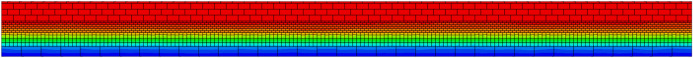
Initial numerical model.

To more accurately reproduce the movement behavior of the roof after coal seam extraction and to investigate the temporal and spatial evolution characteristics of the abutment pressure ahead of the working face, a corresponding numerical model was established using the 3DEC discrete element simulation software. The numerical model is specifically designed to investigate the influence of periodic roof fracture and elastic rebound on abutment pressure evolution ahead of the working face. The model is a two-dimensional horizontal section with geometric dimensions of 350 m × 28 m (length × width), consisting of four main strata: the main roof, immediate roof, coal seam, and floor. Displacement constraints were applied at the model boundaries, and the mechanical behavior of the rock mass followed the Mohr–Coulomb strength criterion. The strike length of the working face was set to 200 m, with a mining depth of 800 m, a coal seam thickness of 7.2 m, a main roof thickness of 9.9 m, and an immediate roof thickness of 5.9 m. The primary objective of this numerical model is to analyze the roof movement phenomena and the macroscopic variation characteristics of advanced support pressure during the coal seam mining process. Therefore, a two-dimensional plane strain model was selected, with the measurement line for advanced support pressure arranged within the coal seam to enable real-time tracking of pressure distribution and variations. The numerical calculation model, after the division of blocks and elements, is shown in Fig 2. The coal-rock mechanical parameters of the simplified geological layers of a specific mine model are listed in Table 1. After the model was established, a static equilibrium calculation was first performed to ensure that the initial state of the model was in equilibrium, followed by the simulation of coal seam extraction. Once the static equilibrium calculation was completed, the coal mining process was simulated, starting from the left side of the model, modeling the roof deformation, displacement, and changes in advanced support pressure during the mining process.

**Table 1:** Mechanical parameters of materials used in the model.

Material	Density (kg·m ⁻ ³)	Elastic Modulus (GPa)	Poisson’s Ratio	Cohesion (MPa)	Internal Friction Angle (°)	Tensile Strength (MPa)
Main roof	2707	20.85	0.3	11.94	30.68	3
Immediate roof	2650	21.6	0.11	3.2	32	2.5
Coal seam	1430	3.4	0.16	7	35	1.6
Floor	2650	10.6	0.13	8.5	32	2.7

Although the present study focuses on a two-dimensional mechanical response along the mining direction, the 3DEC was adopted to ensure accurate representation of block interaction, contact force transmission, and fracture evolution. A quasi-two-dimensional plane strain configuration was imposed by restricting out-of-plane deformation and applying displacement constraints, allowing the model to capture essential three-dimensional mechanical mechanisms while maintaining two-dimensional stress characteristics.

### 2.2. Numerical simulation results and analysis

**Fig 3:**
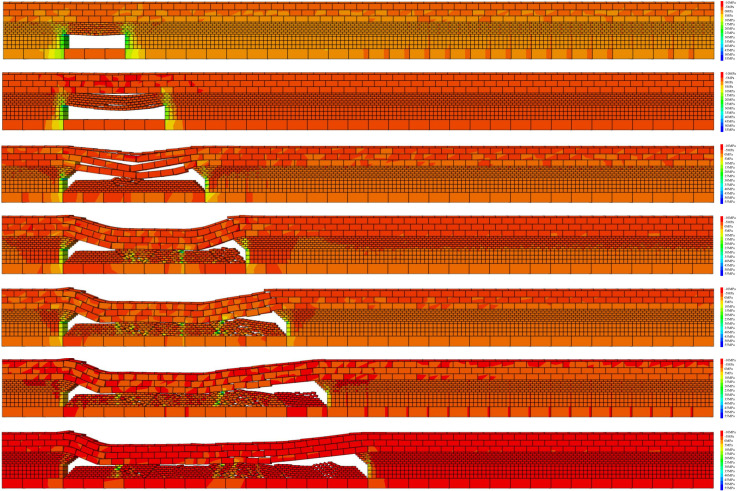
Vertical stress distribution contour of the surrounding rock in the mining face.

The spatiotemporal evolution of the abutment pressure distribution under the influence of the roof fracture–rebound behavior was analyzed. The migration characteristics of the roof strata and the distribution of the abutment pressure at different time steps are shown in Fig 3.

**Fig 4:**
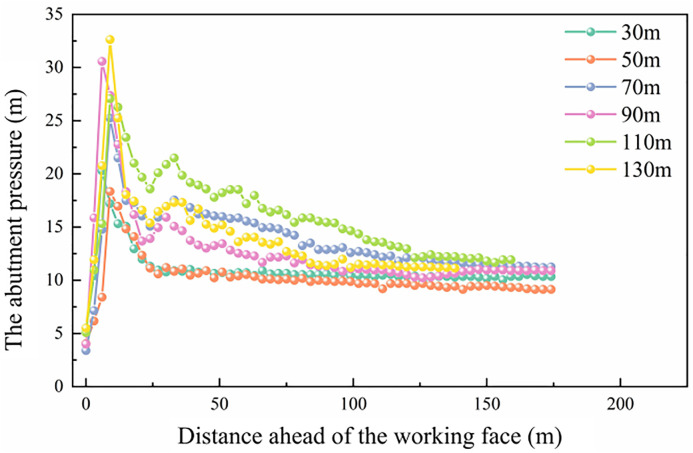
Distribution characteristics of the abutment pressure ahead of the working face.

According to Fig 3, when the working face advances 30 m, the roof has not yet deformed, and a stress concentration appears ahead of the face. When the working face advances to 50 m, delamination occurs between the immediate roof and the main roof, accompanied by the development of vertical fractures in the immediate roof and its downward bending and subsidence. At an advance of 70 m, the immediate roof undergoes its first fracture with a fracture length of approximately 32 m. After the initial fracture, a suspended roof section of about 20 m is formed, and the peak abutment pressure reaches 27.43 MPa. When the working face advances to 90 m, the roof fractures again, with a fracture length of about 15 m and a suspended roof section of approximately 20 m. During this stage, the stress concentration zone in the goaf expands, and the peak abutment pressure increases to 31.66 MPa. At 110 m, another roof collapse occurs, with a periodic weighting interval stabilizing at approximately 20 m, forming a suspended roof of 20 m in length, and the peak abutment pressure reaching 26.95 MPa. When the working face advances to 130 m, another periodic roof collapse takes place, producing a 25 m suspended roof, with the peak abutment pressure rising to 32.67 MPa. As the working face continues to advance to 150 m, the range of the stress concentration zone in the surrounding rock of the goaf gradually expands, and the roof fracture step distance remains stable between 20 m and 25 m. The distribution of abutment pressure also becomes relatively stable. At this stage (150 m), the statistical analysis of the abutment pressure and its influence range during coal seam extraction is shown in Fig 4.

**Fig 5:**
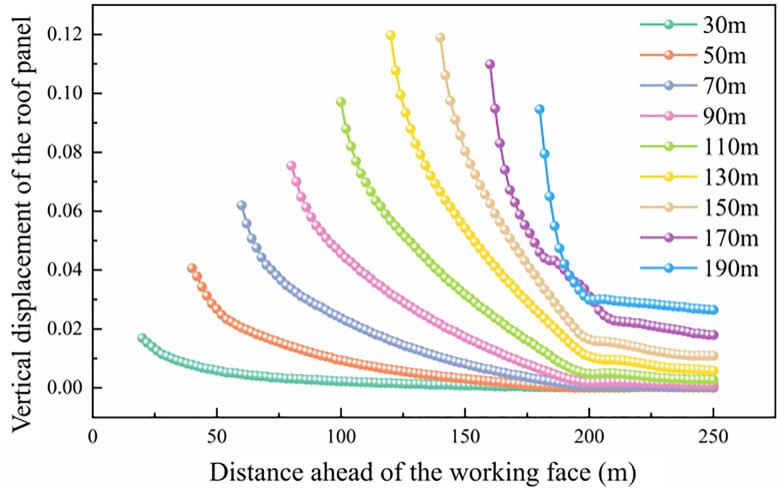
Vertical displacement of the roof during working face advancement.

When the working face was advanced by 30 m, the peak abutment pressure reached 20.21 MPa. As the face advanced to 50 m, the peak value decreased to 18.45 MPa, while at 70 m, it increased again to 27.43 MPa. When the working face advanced to 130 m, the peak abutment pressure reached its maximum value of 32.55 MPa, with the maximum width of the plastic zone reaching 8.6 m and the overall influence range of the abutment pressure extending up to 106 m. In addition, at the early excavation stages (10 m and 30 m), the abutment pressure distribution curve exhibited the conventional first pressure-relief zone. After the initial breakage of the main roof and the transition into the periodic weighting stage, a certain length of cantilever roof was formed. Consequently, a stress reduction zone appeared approximately 23 m ahead of the working face. Unlike the conventional “pressure-relief–loading” distribution pattern, the abutment pressure in this longwall face exhibited a distinctive “pressure-relief–loading–pressure-relief” distribution. The stress field ahead of the coal wall could be divided into four regions: the stress reduction Zone I, the stress concentration zone, the stress reduction Zone II (secondary pressure-relief zone), and the original in-situ stress zone. During the mining process, substantial elastic energy accumulated in the roof strata. When the roof used the coal wall as a pivot, elastic rebound (roof contraction) occurred in the area ahead of the working face. This behavior led to a partial reduction in abutment pressure between the pressure peak and the original stress zone, forming the secondary pressure-relief zone. The secondary stress-relief zone (Zone II) is identified based on the local reduction of vertical stress between the abutment pressure peak. The vertical displacement of the roof induced by the roof fracture and elastic rebound during the working face advancement is shown in Fig 5.

As shown in Fig 5, the vertical displacement curve of the hard roof during the working face advance indicates that, with the progressive extraction of the coal seam, the subsidence of the hard roof at the coal wall gradually increases. When the coal seam is mined up to 140 m, the maximum vertical displacement of the hard roof at the coal wall reaches 120 mm. As the working face continues to advance toward the stop line, the vertical displacement of the hard roof at the coal wall gradually decreases. According to Fig 5, when the coal seam is mined up to 160 m and 180 m, the hard roof exhibits an upward displacement of approximately 21 m ahead of the working face, indicating an upward bending (anti-arch) deformation of the roof in this region. At different mining distances, the moment of roof fracture is accompanied by a noticeable elastic rebound behavior. Consequently, the vertical displacement of the main roof at the coal wall exhibits a variation trend that is highly consistent with the evolution of the abutment pressure in front of the working face. The numerical simulation results of stress redistribution and roof fracture behavior serve as the mechanical basis for the subsequent analysis of gas pressure evolution and outburst triggering mechanisms.

## 3. Mechanism analysis of gas outburst induced by roof fracture based on numerical results

### 3.1. Influence of roof elastic rebound on gas emission from the working face

After the roof fracture, its elastic rebound behavior causes deformation and the development of fractures in the coal seam. The formation of the secondary stress relief zone (Zone II) leads to the reopening of fractures within the coal mass, which directly affects the gas flow capacity in the coal seam. The energy stored in the roof and the energy released from gas emission jointly act on the coal seam or the goaf, potentially inducing abnormal gas emission or even coal and gas outburst events. The expression for the gas emission energy is given by [28]:


$$W=\text{1000}\frac{p_{\text{0}}V_{\text{0}}}{n-\text{1}}\left|{\left(\frac{p_{\text{rs}}}{p_{\text{0}}}\right)}^{\frac{n-\text{1}}{n}}-\text{1}\right|$$ (5)

In the equation, *P₀* denotes the expansion energy of the free gas, *V₀* represents the fracture volume within the coal body, and *n* is the adiabatic index.

**Fig 6:**
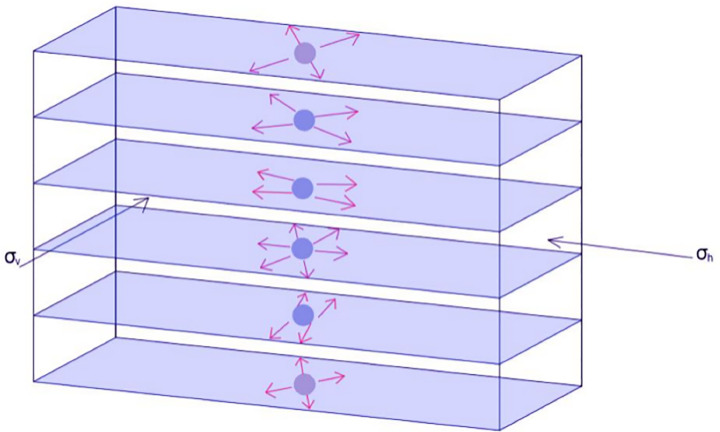
Crack propagation direction in coal under stress.

As the working face advances, the stress peak gradually migrates forward, and the coal structure undergoes continuous damage under cyclic stress loading, resulting in dynamic changes in both porosity and permeability, as illustrated in Fig 6. When the ratio of horizontal to vertical stress exceeds 1, the maximum principal stress is dominated by the horizontal component, and the horizontal stress is significantly greater than the vertical stress. Under such conditions, coal fractures primarily propagate along the horizontal direction, and as this ratio increases, the cracks tend to rotate toward the vertical direction. When the horizontal stress equals the vertical stress, the stress field becomes uniform, leading to isotropic fracture propagation in all directions. Conversely, when the horizontal stress is less than the vertical stress (i.e., the ratio is less than 1), the maximum principal stress is mainly influenced by the vertical component, and fractures extend predominantly in the vertical direction. Therefore, the stress state not only affects the pore structure of the coal but also governs its fracture propagation behavior. The transformation of the stress regime determines whether fracture extension occurs preferentially in the vertical or horizontal orientation. This structural evolution directly controls the gas migration pattern and its flow velocity within the coal seam. According to both measured data and numerical simulations, the pressure term P₀ in the energy expression is strongly influenced by the abutment pressure of the mining field and exhibits distinct periodic fluctuations corresponding to the periodic weighting behavior of the roof. Consequently, the gas flow velocity and emission rate also display periodic variations over time. This phenomenon indicates that the elastic rebound behavior of the roof, through cyclic stress perturbations and fracture evolution effects, fundamentally governs the dynamic response mechanism of gas migration in the coal seam.

With the repeated occurrence of periodic roof weighting, the coal body undergoes multiple cycles of stress loading and unloading, during which part of its stored elastic energy is released, leading to a gradual reduction in residual elastic energy. During the repeated “pressure relief—gas release—pressure relief” process of coal and gas outbursts, pressure undergoes periodic variations. These periodic fluctuations are the primary driving force controlling coal body deformation and gas migration. Therefore, coal and gas outbursts are essentially the result of multi-field coupling among stress, energy, and gas flow. The periodic weighting of the roof regulates the spatiotemporal distribution of mining-induced stresses, thereby indirectly controlling the coal permeability, gas flow velocity, and gas emission rate, which in turn determine the intensity and frequency of coal and gas outbursts. The elastic rebound and periodic weighting behavior of the roof play a crucial role in determining both the temporal characteristics of energy release from the coal mass and the conditions leading to outburst initiation.

### 3.2. Influence of roof elastic rebound on coal and gas outbursts in the working face

**Fig 7:**
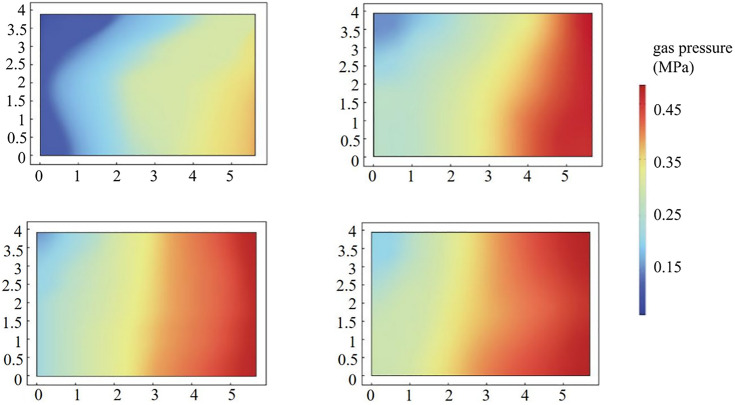
Numerical simulation of gas pressure at the instant of roof fracture (50m).

To elucidate the gas pressure variation induced at the instant of roof fracture, a numerical model was established using COMSOL Multiphysics, in which gas flow was governed by Darcy’s law. The instantaneous roof fracture was represented by applying a transient unloading boundary condition to the coal body. The seepage parameters used in the simulation are listed in Table 2. The gas pressure variation at a mining advance of 50 m is shown in Fig 7.

**Table 2:** Seepage parameters.

Parameter	Value
Porosity	0.08
Fracture porosity	0.045
Permeability (mD)	0.12
Gas dynamic viscosity (Pa·s)	1.08e-5
Diffusion coefficient (m^2^/s)	5.6e-12
Pore pressure coefficient (α)	1.00

As shown in Fig 7, roof fracture and the associated elastic rebound behavior constitute a key dynamic driving force for coal and gas outbursts by inducing abrupt stress unloading, enhanced permeability, and rapid gas pressure release. The gas pressure evolution exhibits pronounced transient characteristics, which clearly distinguish outburst-related gas behavior from conventional gas seepage under routine mining conditions and provide a quantitative explanation for the sudden and violent nature of coal and gas outbursts following roof fracture. Owing to the formation of the second pressure-relief zone, together with the combined effects of localized stress concentration and gas compression, a short-term increase in gas pressure may occur within the coal seam. Subsequently, as fracture connectivity is enhanced and permeability increases abruptly, the gas pressure evolution induced by roof fracture can be divided into three distinct stages: (i) a pre-fracture quasi-equilibrium stage, (ii) a short-term gas pressure accumulation stage, and (iii) a rapid pressure relief and release stage.

The stress changes in the coal seam during the mining process lead to coal body damage, and the evolution of this damage further affects gas flow. To quantitatively describe this coupled relationship, a damage mechanics model is used to simulate the interaction between stress and the damage field.


$$\frac{\partial \sigma }{\partial t}=C(\epsilon ,D)$$ (6)

Where *σ* represents stress, *∊* represents strain, *D* is the damage variable, and *C(∊, D)* denotes the coupling relationship between stress and the damage variable. The accumulation of damage leads to changes in the permeability of the coal seam, thereby affecting gas flow. There exists a strong coupling relationship between gas flow and the stress field. Stress disturbances within the coal seam influence the flow path and velocity of gas, while gas flow, in turn, alters the permeability of the coal seam, which subsequently impacts the distribution of the stress field. The coupled equations for gas flow and the stress field are as follows:


$$\frac{\partial P}{\partial t}=-\nabla ∙(q)+f(\sigma ,D)$$ (7)

Where *P* represents gas pressure, *q* represents gas flow, and *f(*σ, *D)* is the function that describes the influence of stress and damage on gas flow. Using the above coupling equations, the interaction between stress disturbance, damage field evolution, and gas flow during the coal seam mining process is quantitatively analyzed. The study shows that with the increase in mining depth and the intensification of stress disturbances, the local concentration of gas pressure in the coal seam becomes more pronounced. At the initial moment, there is no induced damage in the coal seam, the fractal dimension of fractures is the initial value *D*_*f0*_, and the strain softening parameter is 0. As coal body damage continues to develop, the coal body exhibits plastic flow behavior. At this point, the strain softening parameter reaches its critical value, and the fractal dimension is *D*_*fr.*_ The fractal dimension evolving with strain softening during fracture can be expressed as [29]:


$$D_{f}=D_{f_{\text{0}}}+(D_{f}-D_{f_{\text{0}}})D$$ (8)

The gas exchange between fractures and coal matrix is regarded as a homogeneous unit, simplifying the gas exchange process between fractures and coal matrix, as well as the dimensions of the fractures and coal matrix. Affected by mining, the fractal dimension of fractures evolves from the initial value *D*_*f0*_ to *D*_*f*_, and the total fracture length increases from *b*_*0*_ to *b*. The fracture volume is relatively small in proportion to the coal body, and the fracture area in this section can be neglected. Therefore, the cross-sectional area is approximately equal to the total area of the coal matrix in the profile. Since the deformed coal body after mining is relatively small compared to the total coal seam volume, the cross-sectional area is considered to be a constant. Consequently, the dimensions of the coal matrix are expressed as follows:


$$a=\frac{D_{f\text{0}}}{D_{f}}\frac{D_{f}-\text{1}}{D_{f\text{0}}-\text{1}}r_{\text{0}}^{D_{f}-D_{p}}a_{\text{0}}$$ (9)

In the equation, *D*_*f0*_ represents the fractal dimension of the original coal body fractures, *D*_*f*_ denotes the fractal dimension of the damaged coal body fractures, and *r*_*0*_ is the minimum fracture length in millimeters. From equation (9), the following can be derived:


$$\begin{array}{cc} & \Delta a=a-a_{\text{0}}\\ & =\left(\frac{D_{f\text{0}}}{D_{f}}\frac{D_{f}-\text{1}}{D_{f\text{0}}-\text{1}}r_{\text{0}}^{D_{f}-D_{f\text{0}}}-\text{1}\right)a_{\text{0}} \end{array}$$ (10)

Therefore, the deformation expression of the matrix width under damage conditions is as follows:


$$\frac{∆a}{a_{\text{0}}}=\frac{D_{f\text{0}}}{D_{f}}\frac{D_{f}-\text{1}}{D_{f\text{0}}-\text{1}}r_{\text{0}}^{D_{f}-D_{f\text{0}}}-\text{1}$$ (11)

The mechanical properties of coal deteriorate with the increase in stress, leading to changes in the elastic modulus. Typically, the variation in elastic modulus is used to represent the damage variable. According to the theory of elastic damage, the relationship between the elastic modulus and the damage variable can be expressed as:


$$E=(\text{1}-D_{m})E_{\text{0}}$$ (12)

In the equation, *E* represents the elastic modulus (MPa), *E*_*0*_ is the initial elastic modulus (MPa), and *D*_*m*_ denotes the mechanical damage variable. The amount of gas desorption from the coal body at the moment of roof fracture in the S_5_ working face of a specific mine is shown in Table 3.

**Table 3:** Gas desorption from the coal body at the moment of roof fracture.

Detection point No.	Sampling location (m) (distance from the working face)	Gas desorption before fracture (m³/t)	Gas desorption after fracture (m³/t)
1	15	6.3215	3.7531
2	35	5.9883	3.6014
3	55	6.1524	3.5324
4	75	6.0832	3.1104
5	95	5.7734	3.6151
6	115	6.2157	3.9172
7	125	5.8649	3.7608
Average	–	6.0571	3.6129

**Fig 8:**
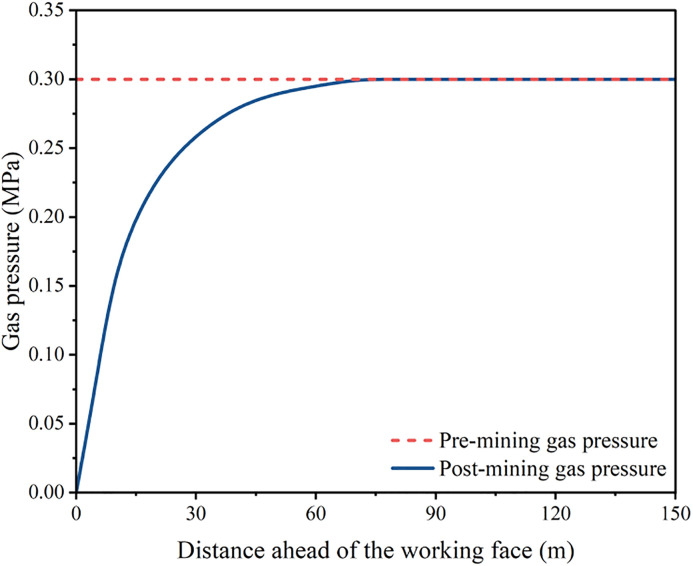
Gas pressure distribution before and after periodic roof weighting.

**Fig 9:**
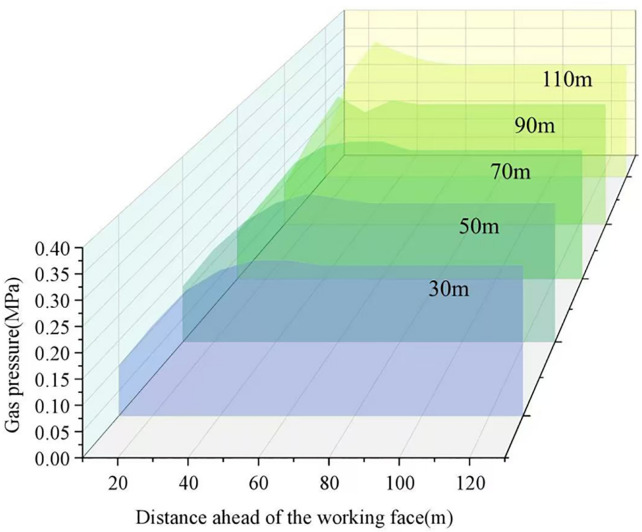
Variation of gas pressure under different mining distances.

As the working face advances, the span of the roof rock beam gradually increases. When the span reaches its critical limit, the roof beam fractures and collapses, marking the onset of a periodic weighting event. This process repeats cyclically, generating dynamic disturbances associated with periodic roof weighting. The gas pressure distributions before and after periodic roof weighting are shown in Fig 8, while the variations in gas pressure under different mining distances are presented in Fig 9.

As shown in Fig 8, before coal seam extraction, the gas within the seam remains in a dynamic equilibrium state, and the gas pressure exhibits a uniform distribution throughout the coal seam. During the mining process, this equilibrium is disrupted, leading to nonequilibrium gas migration within the coal body. Consequently, localized zones of gas pressure concentration and reduction emerge in the coal mass ahead of the working face. The degree of gas pressure concentration increases progressively with the intensity of mining-induced disturbance. In the near-field region of the working face—particularly within the coal seam adjacent to the face—the gas pressure decreases sharply, reflecting the strong influence of stress redistribution and permeability enhancement induced by roof fracture and elastic rebound.

As shown in Fig 9, after a roof fracture, the elastic rebound of the roof causes a sudden change in the stress structure of the coal mass ahead of the working face. This results in intense deformation, localized fracturing, and displacement within the coal body, which in turn promotes rapid gas desorption and instantaneous release. At different mining distances, the gas pressure within the coal seam in front of the working face evolves from an initial dynamic equilibrium state to a pressure rise phase, followed by a rapid pressure drop phase. Overall, the evolution of gas pressure can be divided into three distinct stages: The initial dynamic equilibrium stage, the pressure increase stage, and the rapid pressure reduction stage. The gas pressure in the coal seam ahead of the working face first gradually increases and then decreases sharply, exhibiting a cyclic and forward-migrating pattern with the progression of mining. After the roof fractures, the coal near the face moves more rapidly, and the peak stress zone shifts forward. Within approximately 50 m ahead of the face, the coal movement velocity gradually decreases, indicating that the second pressure-relief zone expands progressively from the fracture line into the deeper coal mass. The periodic pressure fluctuations induced by roof fracture continuously disrupt the original structure of the coal body, leading to cyclic variations in porosity and permeability. High-pressure gas initially stored within micropores rapidly expands and migrates through newly formed fractures and fault channels. Due to coal fragmentation, the effective gas-acting surface area increases by nearly elevenfold, from the pore scale to the exposed coal surface. Under the action of elevated gas pressure, the fragmented coal is rapidly ejected, forming a coupled “gas-driven coal ejection” effect, which ultimately leads to the occurrence of coal and gas outbursts.

In summary, a coal and gas outburst is a dynamic energy release disaster caused by the instantaneous release of accumulated energy in localized coal masses under the combined action of high stress and high gas pressure. The roof fracture and elastic rebound behaviors during mining serve as key dynamic sources that trigger this process. The elastic rebound of the roof not only alters the mechanical equilibrium and fracture structure of the coal body but also introduces time-dependent coupling effects between gas release and migration. The periodic energy release of the roof produces a significant dynamic coupling relationship among coal stress, fracture evolution, and gas occurrence state, providing both an essential driving mechanism and a diagnostic indicator for coal and gas outbursts.

## 4. Conclusions

(1) A new distribution feature of advanced abutment pressure under hard roof suspension conditions was identified. As the working face advances, the suspension length of the main roof increases. The elastic rebound behavior during roof fracture reduces the vertical stress acting on the coal seam, thereby forming a second pressure-relief zone in front of the working face.
(2) The influence of different suspension lengths on the advanced abutment pressure distribution was investigated. The corresponding displacement and pressure distribution curves indicate that a longer suspended roof leads to a higher peak abutment pressure, further validating the existence of the second pressure-relief zone.
(3) With increasing main roof suspension length, the degree of stress concentration continues to intensify, and both the extent and magnitude of the second pressure-relief zone expand. When the main roof fractures, localized rebound and contraction occur in the roof strata ahead of the fracture line, causing a reduction in stress concentration and a partial recovery of abutment pressure within the second pressure-relief zone.
(4) The elastic rebound and periodic weighting behaviors of the roof at the moment of fracture play a crucial role in determining the conditions of energy release and gas outburst occurrence. Roof fracture and elastic rebound during mining are thus key dynamic driving mechanisms for coal and gas outburst initiation.

## Supporting information

S1 File Data. (PDF)
