# Supplementary material for: Study on the influence of periodic roof fracturing on the mechanical disturbance characteristics of coal and gas outburst behavior in the mining face
Source: PLoS One. 2026 Feb 13;21(2):e0337026. doi: 10.1371/journal.pone.0337026 (PMC12904452; doi:10.1371/journal.pone.0337026)
Supplement: S1 File — (PDF) [file pone.0337026.s001.pdf]

Fig.4

0	5.51038	4.05709	3.391	3.99654	5.08651	5.51038
3	10.4152	6.17647	7.14533	15.8651	10.9602	11.9291
6	20.2855	8.41696	14.7751	30.5796	15.3201	20.7699
9	17.1972	18.3478	25.2509	27.3702	27.0675	32.6384
12	15.3201	16.955	21.4965	22.7682	26.2803	25.2509
15	15.0779	14.8356	17.5	18.3478	23.4343	18.045
18	12.9585	14.109	17.3183	16.1678	21.0121	17.4394
21	11.9896	12.3529	16.0467	13.6851	19.6799	16.5917
24	11.3235	11.1419	15.0779	13.9273	18.59	15.3806
27	10.9602	10.5969	15.9256	14.9567	20.1038	16.4706
30	10.7785	11.2024	16.955	15.9256	20.891	16.955
33	10.8997	10.8391	17.5606	15.0779	21.4965	17.3183
36	10.8391	11.0813	17.3183	14.654	19.8616	17.3183
39	11.0208	10.4758	16.8339	13.7457	19.1955	15.6228
42	10.7785	10.6574	16.2284	13.3218	18.9533	16.7128
45	10.8997	10.8997	16.2284	13.019	18.59	15.2595
48	10.6574	10.2336	16.0467	13.2612	17.8028	14.8962
51	10.718	10.7785	15.9862	13.4429	18.2266	15.199
54	10.5969	10.2941	15.8045	12.8374	18.5294	14.5934
57	10.718	10.4152	15.8651	12.5346	18.5294	13.6246
60	10.718	10.5363	15.5623	12.4135	17.1972	14.0484
63	10.4758	10.3547	15.3806	12.2924	17.9844	14.0484
66	10.8997	10.1125	14.9567	11.6869	16.7734	13.564
69	10.5969	10.1125	14.9567	12.1713	16.41	13.3824
72	10.6574	10.0519	14.8356	12.1713	16.5917	13.6246
75	10.6574	10.1125	14.4723	12.2924	16.1678	12.7163
78	10.5363	9.99135	14.2301	11.6263	15.4412	12.474
81	10.5363	10.173	13.2612	11.9291	15.8651	12.2924
84	10.5363	9.87024	13.5035	11.4446	15.8651	11.6263
87	10.5363	9.99135	12.8979	11.3235	15.5623	11.4446
90	10.5969	9.9308	12.8979	11.3235	15.4412	11.3841
93	10.4758	9.87024	12.8979	11.2024	15.3806	11.5052
96	10.718	9.9308	13.0796	10.8391	14.8356	11.9896
99	10.4152	9.87024	12.5952	11.0813	14.654	11.2024
102	10.4758	9.68858	12.7163	11.0813	14.3512	11.5052
105	10.4758	9.74913	12.5952	11.0208	13.8062	11.4446
108	10.3547	9.68858	12.4135	10.9602	13.6246	11.5657
111	10.4758	9.20415	12.1713	10.9602	13.564	11.4446
114	10.5363	9.68858	12.2924	10.7785	13.2612	11.4446
117	10.3547	9.68858	11.808	10.718	13.1401	11.3235
120	10.4152	9.68858	12.1107	10.5363	12.9585	11.263
123	10.4152	9.50692	12.1107	10.2941	12.1107	11.263
126	9.87024	9.68858	12.1107	10.2336	12.2318	11.263

129	10.3547	9.50692	11.8685	10.2941	12.4135	11.263
132	10.3547	9.44637	11.808	10.4152	12.2318	11.2024
135	10.3547	9.32526	11.6869	10.5969	12.2318	11.2024
138	10.2941	9.44637	11.7474	10.6574	12.2318	11.1419
141	10.3547	9.1436	11.6263	10.8391	12.1107	
144	10.3547	9.44637	11.5052	10.8997	12.0502	
147	10.2941	9.44637	11.5052	11.0208	12.1107	
150	10.173	9.50692	11.3841	10.9602	11.808	
153	10.3547	9.44637	11.3841	10.9602	11.6869	
156	10.0519	9.38581	11.4446	10.9602	11.9291	
159	10.3547	9.32526	11.3235	10.8997	11.9291	
162	10.3547	9.32526	11.3235	10.8997		
165	10.5363	9.20415	11.3235	10.8997		
168	10.4152	9.1436	11.263	10.9602		
171	10.3547	9.1436	11.2024	10.8997		
174	10.3547	9.1436	11.263	10.8997		

Fig.5

20	0.01688				
22	0.01534				
24	0.01398				
26	0.01257				
28	0.01146				
30	0.01073				
32	0.01				
34	0.00934				
36	0.00882				
38	0.00825				
40	0.00786	0.04066			
42	0.00737	0.03788			
44	0.00687	0.03436			
46	0.00656	0.03126			
48	0.00628	0.02861			
50	0.0059	0.02676			
52	0.0056	0.0248			
54	0.00519	0.02327			
56	0.00514	0.02242			
58	0.00493	0.02136			
60	0.00466	0.02045	0.06201		
62	0.00442	0.01976	0.05578		
64	0.00424	0.01909	0.0507		
66	0.00412	0.01825	0.04756		
68	0.00397	0.01772	0.0443		
70	0.00385	0.01703	0.04175		
72	0.00355	0.01637	0.04008		
74	0.00354	0.01582	0.03822		
76	0.00336	0.01525	0.03649		
78	0.00318	0.01462	0.03486		
80	0.00315	0.01406	0.03366	0.07547	
82	0.00303	0.01357	0.03242	0.06999	
84	0.00302	0.01306	0.0311	0.06486	
86	0.00281	0.01249	0.03026	0.06131	
88	0.00275	0.01203	0.02918	0.05798	
90	0.00275	0.01153	0.02813	0.05512	
92	0.00259	0.01102	0.02745	0.0531	
94	0.00247	0.01069	0.02643	0.05092	
96	0.00244	0.0103	0.0254	0.04899	
98	0.00232	0.00967	0.02462	0.04743	
100	0.0022	0.0094	0.02375	0.04569	0.09709
102	0.0022	0.00907	0.02278	0.04404	0.08798
104	0.0021	0.00865	0.02212	0.04278	0.082

106	0.00204	0.00832	0.02122	0.04124	0.07695				
108	0.00192	0.00799	0.02041	0.0396	0.07279				
110	0.00192	0.00769	0.01973	0.03848	0.06978				
112	0.00188	0.00739	0.01892	0.03703	0.06661				
114	0.00176	0.00709	0.01814	0.03556	0.06351				
116	0.00164	0.00678	0.01756	0.0346	0.06159				
118	0.00164	0.00645	0.01686	0.03325	0.05898				
120	0.00164	0.00628	0.01609	0.03189	0.05679	0.11973			
122	0.00154	0.00601	0.01554	0.03087	0.05495	0.10774			
124	0.00148	0.00577	0.01485	0.02985	0.05299	0.0995			
126	0.00136	0.00549	0.0142	0.02862	0.05119	0.09347			
128	0.00136	0.00528	0.01366	0.02762	0.04959	0.08797			
130	0.00136	0.00501	0.01312	0.02651	0.0477	0.08292			
132	0.00133	0.00484	0.01267	0.02543	0.04578	0.07923			
134	0.0012	0.00459	0.01198	0.02459	0.04421	0.07553			
136	0.00108	0.00441	0.01149	0.02351	0.04235	0.07204			
138	0.00108	0.0042	0.01095	0.02252	0.0406	0.06931			
140	0.00105	0.00393	0.01041	0.02167	0.03919	0.06662	0.11892		
142	0.00107	0.00379	0.00997	0.02056	0.03739	0.06369	0.10618		
144	0.00107	0.00366	0.00949	0.01975	0.03591	0.06142	0.0975		
146	9.20551E-4	0.00339	0.00898	0.01908	0.03462	0.05904	0.09109		
148	7.98543E-4	0.00324	0.00856	0.01811	0.03312	0.05643	0.08576		
150	7.96386E-4	0.00312	0.00822	0.01725	0.03165	0.05413	0.08033		
152	7.94229E-4	0.00285	0.00771	0.01637	0.03044	0.05184	0.07604		
154	7.92071E-4	0.00273	0.00732	0.01568	0.029	0.04948	0.07247		
156	7.89913E-4	0.00258	0.0069	0.01484	0.02759	0.04732	0.0688		
158	6.37944E-4	0.00243	0.00648	0.01412	0.0265	0.04525	0.06561		
160	6.35786E-4	0.00231	0.00628	0.01327	0.02515	0.04307	0.06259	0.10988	
162	5.13779E-4	0.00204	0.00582	0.01261	0.02374	0.04117	0.05922	0.09482	
164	5.11622E-4	0.00201	0.00543	0.01189	0.02271	0.03918	0.05635	0.08307	
166	3.89637E-4	0.00177	0.00513	0.01108	0.02136	0.03721	0.0537	0.07407	
168	5.07307E-4	0.00162	0.00474	0.01033	0.02001	0.03552	0.05071	0.06716	
170	5.0515E-4	0.0015	0.00447	0.00976	0.01902	0.03375	0.04811	0.06303	
172	5.02487E-4	0.00135	0.00419	0.00904	0.01784	0.03181	0.04581	0.05884	
174	3.51022E-4	0.00111	0.00387	0.00834	0.01661	0.03022	0.04313	0.05532	
176	3.48865E-4	0.00102	0.00345	0.00777	0.01568	0.02851	0.04068	0.05249	
178	2.26857E-4	9.60961E-4	0.00312	0.00708	0.01457	0.02668	0.03843	0.04928	
180	2.247E-4	8.08324E-4	0.00288	0.00645	0.01328	0.02522	0.0359	0.04607	0.09459
182	2.22543E-4	6.90691E-4	0.00258	0.00591	0.01237	0.02354	0.03342	0.04415	0.07946
184	2.20386E-4	6.60914E-4	0.00231	0.00525	0.01108	0.02183	0.03141	0.04325	0.06503
186	2.18229E-4	4.2042E-4	0.00204	0.00462	0.00982	0.02035	0.02895	0.04321	0.05499

188	2.16071E-4	3.89231E-4	0.00177	0.004	0.00868	0.01868	0.02667	0.04191	0.04744
190	2.13258E-4	2.68025E-4	0.0015	0.00355	0.00783	0.01703	0.02451	0.04009	0.04209
192	2.07726E-4	1.5015E-4	0.00123	0.00301	0.00686	0.01533	0.02246	0.03779	0.03826
194	5.9787E-5	1.56783E-6	9.60961E-4	0.00259	0.00608	0.01387	0.02039	0.03615	0.03567
196	5.76294E-5	1.20036E-4	8.09848E-4	0.00231	0.00556	0.01255	0.01849	0.03523	0.03321
198	5.59465E-5	1.2012E-4	6.90691E-4	0.00204	0.00502	0.01132	0.01722	0.03358	0.03124
200	6.65361E-5	1.2012E-4	6.88402E-4	0.00189	0.00487	0.01067	0.01646	0.03108	0.0299
202	6.86926E-5	1.50952E-4	6.90691E-4	0.00189	0.00474	0.0101	0.01593	0.02904	0.02995
204	7.08502E-5	1.8018E-4	6.90691E-4	0.00201	0.00486	0.00981	0.01583	0.02633	0.03005
206	7.30078E-5	1.8018E-4	6.95028E-4	0.00204	0.00501	0.00961	0.0158	0.02459	0.03011
208	7.51644E-5	2.7027E-4	8.10811E-4	0.00204	0.00502	0.00961	0.0156	0.02357	0.02986
210	7.7322E-5	2.7027E-4	8.10811E-4	0.00216	0.00498	0.00959	0.01543	0.02285	0.02961
212	7.94796E-5	2.7027E-4	8.10811E-4	0.00216	0.00475	0.00945	0.01511	0.02258	0.02944
214	8.16362E-5	2.7027E-4	8.10811E-4	0.00216	0.00474	0.00934	0.01482	0.02243	0.02934
216	8.37943E-5	2.7027E-4	7.20209E-4	0.00204	0.00448	0.00907	0.01448	0.02231	0.0291
218	2.35764E-4	2.7027E-4	6.90691E-4	0.00204	0.00436	0.00881	0.01403	0.02204	0.02905
220	2.37922E-4	2.7027E-4	6.90691E-4	0.00195	0.0042	0.00852	0.01372	0.02195	0.02884
222	2.71737E-4	2.7027E-4	6.89667E-4	0.00177	0.00393	0.00819	0.01331	0.02165	0.02876
224	3.62087E-4	2.7027E-4	6.84943E-4	0.00177	0.00387	0.00791	0.01292	0.02129	0.02857
226	3.64243E-4	2.7027E-4	5.40541E-4	0.00177	0.00366	0.00765	0.01265	0.02102	0.02845
228	3.66401E-4	2.7027E-4	4.2042E-4	0.00159	0.00345	0.00718	0.01234	0.02069	0.02828
230	3.68558E-4	2.7027E-4	4.2042E-4	0.0015	0.00339	0.00706	0.01206	0.02042	0.02805

232	3.70716E-4	1.8018E-4	4.2042E-4	0.00159	0.00325	0.00687	0.0118	0.02015	0.02789
234	3.72873E-4	1.8018E-4	4.2042E-4	0.0015	0.00301	0.00664	0.01156	0.01988	0.02772
236	3.7503E-4	1.8018E-4	4.2042E-4	0.0015	0.00312	0.00664	0.01136	0.01961	0.02752
238	3.77187E-4	1.2012E-4	4.2042E-4	0.00135	0.00309	0.00649	0.01123	0.01934	0.0275
240	3.79345E-4	1.2012E-4	4.2042E-4	0.00123	0.00286	0.00637	0.01103	0.01901	0.02718
242	3.82367E-4	1.2012E-4	4.2042E-4	0.00123	0.00285	0.00634	0.01096	0.01853	0.02695
244	5.33472E-4	1.2012E-4	4.2042E-4	0.00123	0.00285	0.00628	0.01095	0.0185	0.02691
246	5.35629E-4	1.2012E-4	4.2042E-4	0.00123	0.00285	0.0061	0.01096	0.01826	0.02673
248	5.37786E-4	1.2012E-4	4.2042E-4	0.00123	0.00285	0.0061	0.01094	0.01799	0.02656
250	5.39938E-4	2.29375E-7	4.2042E-4	0.00123	0.0027	0.00583	0.01093	0.01799	0.02649

Fig.8

0	0.3	0
5	0.3	0.08
10	0.3	0.17
20	0.3	0.23
30	0.3	0.26
40	0.3	0.28
50	0.3	0.29
60	0.3	0.295
70	0.3	0.3
80	0.3	0.3
90	0.3	0.3
110	0.3	0.3
130	0.3	0.3
150	0.3	0.3

Fig.9

10	0.1	0.12	0.11	0.1	0.1
20	0.18	0.2	0.19	0.22	0.28
30	0.25	0.26	0.27	0.32	0.36
40	0.29	0.3	0.31	0.28	0.33
50	0.31	0.32	0.32	0.31	0.31
60	0.31	0.31	0.32	0.3	0.3
70	0.3	0.3	0.3	0.3	0.3
80	0.3	0.3	0.3	0.3	0.3
90	0.3	0.3	0.3	0.3	0.3
110	0.3	0.3	0.3	0.3	0.3
130	0.3	0.3	0.3	0.3	0.3
